# Macrophage Infection via Selective Capture of HIV-1-Infected CD4^+^ T Cells

**DOI:** 10.1016/j.chom.2014.10.010

**Published:** 2014-12-10

**Authors:** Amy E. Baxter, Rebecca A. Russell, Christopher J.A. Duncan, Michael D. Moore, Christian B. Willberg, Jose L. Pablos, Andrés Finzi, Daniel E. Kaufmann, Christina Ochsenbauer, John C. Kappes, Fedde Groot, Quentin J. Sattentau

**Affiliations:** 1 The Sir William Dunn School of Pathology, University of Oxford, South Parks Road, Oxford OX1 3RE, UK; 2 Department of Medicine, Université de Montréal, Montreal, Quebec, H2X 0A9, Canada; 3 Centre de Recherche du Centre Hospitalier de l’Université de Montréal (CRCHUM), Université de Montréal, Montreal, Quebec, H2X 0A9, Canada; 4 The Weatherall Institute of Molecular Medicine, The Nuffield Department of Medicine, The University of Oxford, Headley Way, Oxford OX3 9DU, UK; 5 Servicio de Reumatología, Instituto de Investigación Hospital 12 de Octubre, Madrid 28041, Spain; 6 Department of Microbiology, Infection and Immunology, Université de Montréal, Montreal, Quebec, Canada; 7 The Ragon Institute of MGH, MIT and Harvard, Cambridge, MA 02114, USA; 8 Department of Medicine, University of Alabama at Birmingham, Birmingham, AL 35294, USA

## Abstract

Macrophages contribute to HIV-1 pathogenesis by forming a viral reservoir and mediating neurological disorders. Cell-free HIV-1 infection of macrophages is inefficient, in part due to low plasma membrane expression of viral entry receptors. We find that macrophages selectively capture and engulf HIV-1-infected CD4^+^ T cells leading to efficient macrophage infection. Infected T cells, both healthy and dead or dying, were taken up through viral envelope glycoprotein-receptor-independent interactions, implying a mechanism distinct from conventional virological synapse formation. Macrophages infected by this cell-to-cell route were highly permissive for both CCR5-using macrophage-tropic and otherwise weakly macrophage-tropic transmitted/founder viruses but restrictive for nonmacrophage-tropic CXCR4-using virus. These results have implications for establishment of the macrophage reservoir and HIV-1 dissemination in vivo.

## Highlights

• Macrophages selectively capture and engulf HIV-1-infected T cells
• Uptake of HIV-1-infected T cells drives efficient macrophage infection
• T cell capture is viral Env independent; macrophage infection is Env-receptor dependent
• This represents a route for macrophage infection by transmitted/founder viruses

## Graphical Abstract

**Figure:**
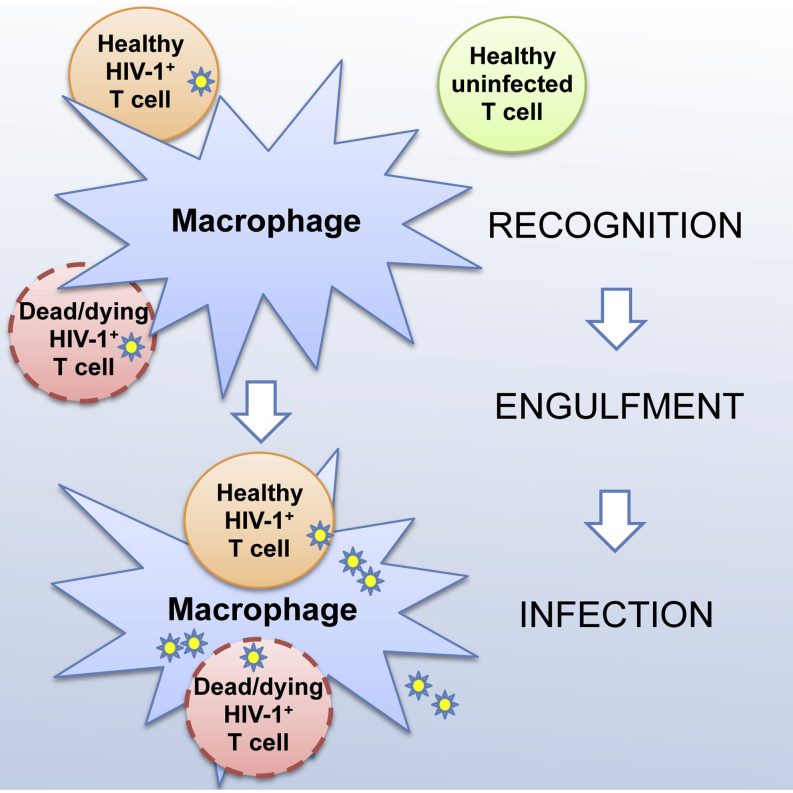

## Teaser

Tissue macrophages play an important role in HIV-1 infection as viral reservoirs. Baxter et al find that engulfment of HIV-1-infected T cells represents an efficient route by which macrophages may become infected. This mode of spread enables weakly macrophage-tropic transmitted/founder viruses, implicated in viral transmission, to infect macrophages.

## Introduction

Macrophages are scavengers that phagocytose dead and dying cells during normal tissue homeostasis, and detect and eliminate infected cells in their role as innate immune sentinels (Devitt and Marshall, 2011, Poon et al., 2010). In immunodeficiency virus-infected hosts, macrophages may comprise up to 10% of infected cells (Zhang et al., 1999), survive for extended periods as a viral reservoir (Gorry et al., 2014), and drive infection-related neurological disorders (Burdo et al., 2013). Tropism of HIV-1 for macrophages is determined both by receptor (CD4) and coreceptor (CCR5 and CXCR4) expression (R5 and X4 viruses, respectively) and by additional less well-defined factors (Duncan and Sattentau, 2011). Viruses transmitted between individuals, termed transmitted/founder (T/F) viruses, are minimally tropic for macrophages (Ochsenbauer et al., 2012, Salazar-Gonzalez et al., 2009), implying that macrophage infection occurs at a late stage after viral transmission when the virus has adapted to infect macrophages more efficiently.

Macrophage infection by cell-free HIV-1 is rate limited by fluid-phase uptake (Carter et al., 2011, Maréchal et al., 2001) and low plasma membrane expression levels of viral entry receptors (Lee et al., 1999). A mode of retroviral infection of CD4^+^ T cells that is more efficient than cell-free spread is cell-to-cell spread (Dale et al., 2013, Sattentau, 2008), exemplified by virological synapses (VSs) and associated structures that drive efficient high-multiplicity infection in vitro (Dale et al., 2013, Sattentau, 2008) and may dominate viral dissemination in vivo (Murooka et al., 2012, Sewald et al., 2012). Infected macrophages transfer high-multiplicity HIV-1 infection to CD4^+^ T cells, promoting reduced viral sensitivity to reverse transcriptase inhibitors and some neutralizing antibodies (Duncan et al., 2013, Duncan et al., 2014, Gousset et al., 2008, Groot et al., 2008). However, the principal mechanism by which HIV-1 infects macrophages is unclear, and the ability of HIV-1-infected T cells to transmit virus to macrophages has not been studied. Since CD4^+^ T cells are proposed to be the major cell type infected by immunodeficiency viruses at transmission and throughout infection (Li et al., 2009, Zhang et al., 1999), we investigated interactions between HIV-1-infected T cells and macrophages to determine whether virus might transfer directly between them. We show that primary monocyte-derived macrophages (MDMs) selectively capture autologous primary HIV-1-infected CD4^+^ T cells, leading to infection of MDMs that is of greater magnitude than the corresponding cell-free virus infection, particularly for T/F viruses.

## Results

### MDM Selectively Capture HIV-1-Infected Healthy and Dying T Cells

**Figure 1:**
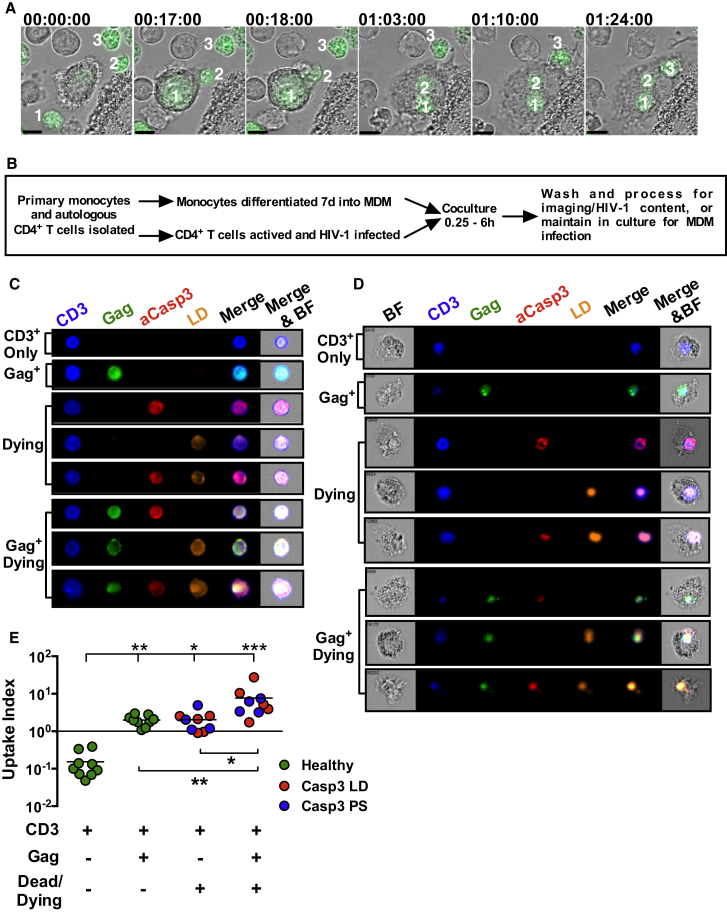
Macrophages Capture and Engulf HIV-1-Infected T Cells (A) Time-lapse sequence of HIV-1-_NL4.3-GFP_-infected Jurkats mixed with MDM. Infected T cells are labeled 1, 2, and 3 in the engulfment order. Scale bar 10 μm. Time shown as hh:mm:ss. See Movies S1, S2, and S3. (B) Experimental strategy for isolation, infection, coculture, and analysis of primary donor cells. (C) ImageStream images of pre-coculture primary HIV-1^+^ CD4^+^ T cells selected for focus, size, and aspect ratio labeled for the phenotypes analyzed: CD3 (T cells, blue), Gag (green), Caspase3 (early apoptosis, red), or live/dead (LD, late apoptosis/necrosis, orange), and all channels merged with and without brightfield (BF). (D) ImageStream images of MDMs associated with T cells selected for focus, MDMs based on size and aspect ratio, then CD3^+^ labeling from 5 × 10^4^ total acquired events showing single engulfed T cells labeled for the different phenotypes analyzed. (E) Summary of data quantified from 10^3^ images such as those in (C) and (D) counted for engulfed T cell phenotype (each circle represents one donor, nine independent donors, three independent experiments). Percentages of MDM containing different subsets of T cells were calculated and expressed as an index based upon the frequency of each T cell subset prior to MDM uptake relative to the frequency after uptake. The third and fourth categories (CD3^+^/dead/dying and CD3^+^/Gag^+^/dead/dying) combine independent experiments reporting markers of cell death: Caspase3 and LD (red) or PS (blue). Statistical analysis using Kruskal-Wallis one-way ANOVA with Dunn’s multiple comparison post hoc test. ^∗^p < 0.05; ^∗∗^p < 0.01; ^∗∗∗^p < 0.001. See Figure S1.

To investigate whether HIV-1-infected T cells might interact with macrophages, we cocultured MDM with CCR5-expressing Jurkat-Tat-CCR5 T cells (Jurkats) or primary CD4^+^ T cells infected with fluorescent X4 (HIV-1_NL4.3-GFP_^+^) or R5 T/F virus (HIV-1_CH077mCherry_^+^) and live-cell imaged over 2 hr. Figure 1A shows stills from Movie S1 (available online), in which a MDM sequentially engulfs three HIV-1_NL4.3/GFP_^+^ Jurkats. Similarly, an MDM engulfs two HIV-1_CH077/mCherry_^+^ Jurkats (Movie S2) or an HIV-1_CH077/mCherry_^+^ primary autologous CD4^+^ T cell (Movie S3). These results suggest that MDM capture is selective for HIV-1^+^ T cells but independent of viral tropism. Since MDMs appeared to ignore apparently healthy, uninfected T cells, we hypothesized that MDM might selectively engulf HIV-1^+^ T cells via direct recognition of cell surface viral antigen and/or indirectly through recognition of T cell death, since HIV-1 infection induces T cell death by apoptosis and other mechanisms (Cooper et al., 2013, Doitsh et al., 2014) and macrophages avidly take up dead and dying cells (Devitt and Marshall, 2011, Poon et al., 2010). We tested this hypothesis using multispectral flow cytometry (ImageStream) quantitation of MDM uptake of HIV-1^+^ and/or dead/dying T cells. An advantage of this technique over conventional flow cytometry is that images can be quantified for capture and internalization of T cells rather than reporting nonspecific cell aggregation or engulfment of cell debris by MDMs. Autologous primary CD4^+^ T cells were isolated, infected with wild-type (WT) R5 HIV-1_BaL_, and processed for imaging as described in Figure 1B. T cells were labeled prior to coculture with MDMs for markers of apoptosis (phosphatidylserine [PS]) or late apoptosis/necrosis (live/dead label [LD]) (Figures 1C and S1A–S1H), and within MDM after coculture, washing, lifting, fixation, and permeabilization for CD3, Gag, and active Caspase3 (Figures 1D and S1I –S1N). Selective MDM uptake of different T cell subsets was quantified by expressing the proportion of each subset in the T cell culture prior to uptake, and within the MDM population subsequent to uptake, as an uptake index (Figures S1I–S1N). An index of <1 indicates T cells are selectively ignored, whereas an index of >1 means T cells are selectively captured from the starting T cell pool. Uninfected (Gag^−^) healthy (Caspase3^−^/PS^−^/LD^−^) T cells were selectively ignored by MDMs compared to either infected/healthy (p < 0.01) or uninfected/dead/dying (p < 0.05) captured T cells (∼10-fold increased capture over uninfected/ healthy). Strikingly, infected/dead/dying T cells were highly significantly captured by MDMs compared to uninfected/healthy (∼50-fold over p < 0.001) and at a significantly higher frequency than either infected/healthy T cells (p < 0.01) or uninfected/dead/dying cells (p < 0.05) (Figure 1E). These data demonstrate that cell death and HIV-1 infection independently promote T cell capture by MDM, but combined they mediate a strong uptake signal.

### Interactions Implicated in MDM Capture of HIV-1-Infected T Cells

**Figure 2:**
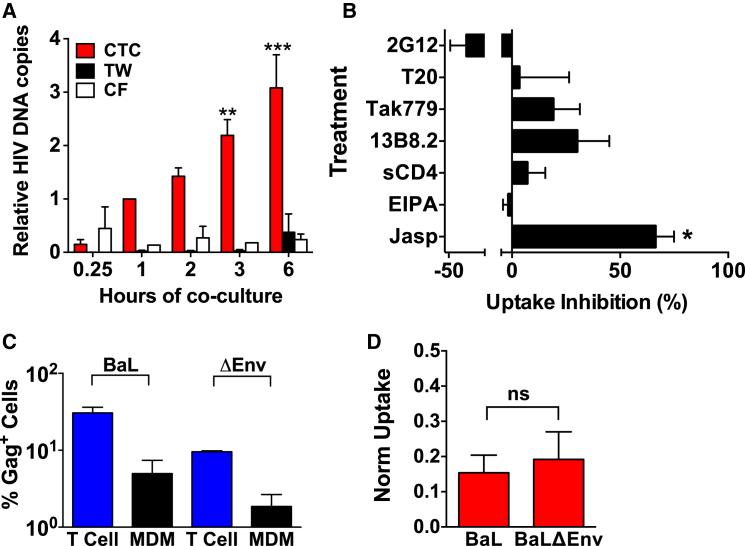
Interactions Implicated in MDM Capture of HIV-1-Infected T Cells (A) Short-term MDM uptake assay of HIV-1_BaL_^+^ primary T cells via direct contact (cell-to-cell, CTC, red), across transwells (TW, black), or HIV-1_Bal_ T cell supernatants added directly to MDM (cell-free, CF, white). Uptake was measured by qPCR for MDM-associated *pol* vDNA, normalized to *β-globin* copy number and to cell-cell transfer at 1 hr = 1. Bars = mean relative HIV-1 DNA copies for five donors + SEM, each donor analyzed in triplicate. ^∗∗^p < 0.01; ^∗∗∗^p < 0.001 by Kruskal-Wallis one-way ANOVA with Dunn’s multiple comparison post hoc test comparing all to CTC T = 0.25. See Figures S2A–S2C. (B) Inhibition of MDM uptake of HIV-1_BaL_^+^ primary T cells. MDMs (Jasp, EIPA, 13B8.2, Tak779, and T20) or HIV-1_BaL_^+^ T cells (2G12, sCD4, and T20) were pretreated for 1 hr with saturating concentrations of inhibitors, cocultured for 30 min, washed to remove unattached T cells, DNA extracted, and qPCR for *pol* vDNA and *β-globin* performed. Bars = mean uptake inhibition (%) for each treatment normalized to appropriate controls + SEM for 4 to 13 independent donors, each analyzed in triplicate. ^∗∗^p < 0.01; ^∗∗∗^p < 0.001 by one-sample t test comparing each treatment to a hypothetical value of 0 (no inhibition) with Sidak’s multiple comparison post hoc test. See Figures S2D–S2F, S3B, and S4. (C and D) Env is not required for HIV-1^+^ T cell uptake. CD4^+^ T cells were synchronously infected for 48 hr with HIV-1_BaL_ or VSV-G pseudotyped HIV-1_BaLΔEnv_ and cultured 1:1 with autologous MDM for 3 hr. MDMs were washed lifted, fixed, permeabilized, and analyzed for intracellular CD3 and Gag by flow cytometry. Bars equal normalized mean of flow cytometry data from two independent donors each analyzed in triplicate + SEM. (C) Percentage Gag^+^ CD4^+^ T cells (blue bars) and MDMs (black bars). (D) Normalized uptake of Gag^+^MDM/Gag^+^ T cells (% Gag^+^ MDM / % Gag^+^ T cells). Bars = normalized mean of flow cytometry data from two independent donors each analyzed in triplicate. ns = p > 0.05, Mann-Whitney U. See Figures S3D and S3E.

To interrogate short-term interactions mediating HIV-1^+^ T cell capture by MDM, we quantified T cell uptake using qPCR of MDM-associated viral (v)DNA (Figure 2A) or luciferase content using the luciferase reporter HIV-1 infectious molecular clone (IMC) HIV-1_BaL-Luc_ (Ochsenbauer et al., 2012). HIV-1^+^ T cells were cocultured with MDM for the times indicated, stringently washed free of unengulfed T cells, and immediately lysed and assayed. We compared HIV-1^+^ T cell uptake with cell-free virus using supernatant derived from the same HIV-1^+^ T cells immediately prior to coculture with MDMs or across 3 μm transwells that prevent T cell transfer but allow free diffusion of virus (Figures S2A–S2C) (Martin et al., 2010). vDNA copy number was normalized to *β-globin* to account for variability in MDM number, and the signal was further normalized to the 1 hr time point to compensate for interdonor variability. We detected increasing T-cell-associated, but not cell-free, HIV-1 signals in MDMs over 6 hr (Figure 2A), consistent with cumulative T cell capture by MDM. To investigate engulfment mechanisms, we blocked actin remodeling, inhibiting cytoskeleton-dependent processes including phagocytosis, or macropinocytosis, using nontoxic or weakly toxic but functional concentrations (Figures S2D–S2F) of jasplakinolide or amiloride (EIPA, Carter et al., 2011), respectively. Jasplakinolide significantly inhibited (p < 0.01) HIV-1^+^ T cell uptake, whereas EIPA did not (Figure 2B), suggesting phagocytosis, rather than macropinocytosis, as a potential uptake mechanism. To further probe uptake interactions we tested inhibitors of HIV-1 envelope glycoprotein (Env)-receptor and cell death receptor engagement. None of the Env-receptor inhibitors (Figures 2B and S3A–S3C) or published inhibitors of interactions driving phagocytosis of dead/dying cells (Figure S4) reproducibly and significantly blocked HIV-1^+^ T cell uptake. We further tested the requirement for Env-receptor interactions by assaying uptake of CD4^+^ T cells infected with WT or Env-deficient HIV-1. Both populations were equivalently captured (Figures 2C, 2D, S3D, and S3E), confirming that HIV-1^+^ T cell-MDM recognition is Env-receptor independent and therefore not mediated via conventional VS signals (Jolly et al., 2004, Sattentau, 2008).

### Capture of HIV-1-Infected T Cells Drives MDM Infection

**Figure 3:**
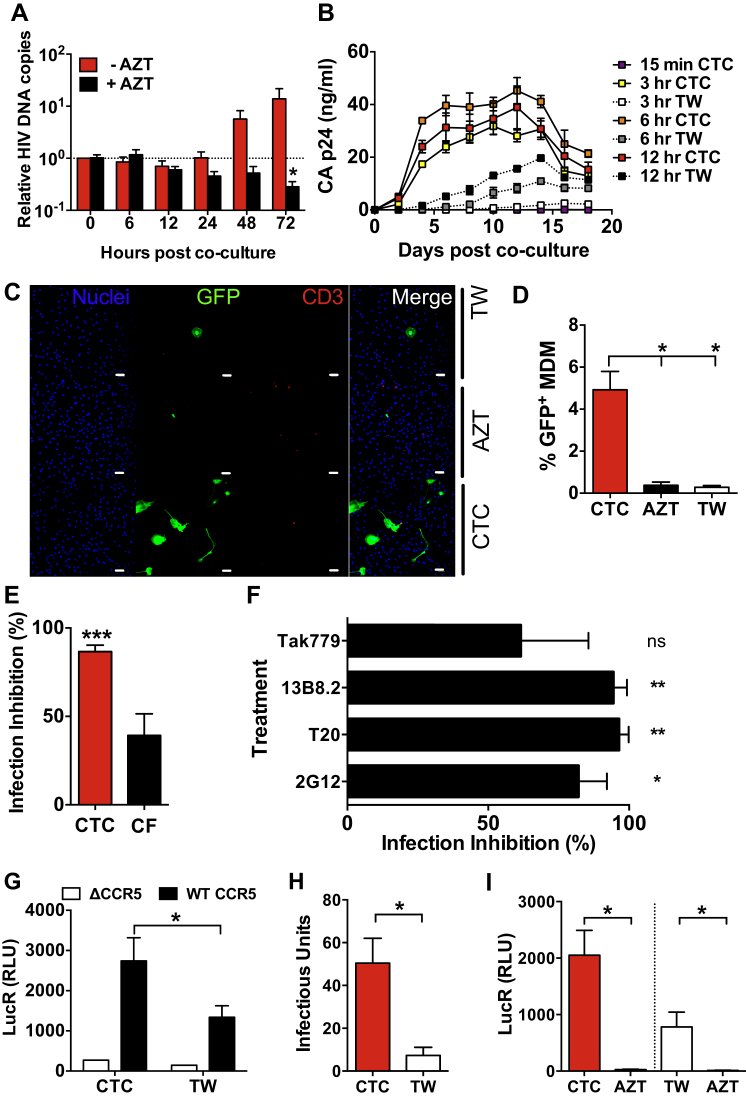
MDM Are Efficiently Infected by HIV-1^+^ T Cells (A) MDM infection by capture of HIV-1^+^ T cells. HIV-1_BaL_^+^ primary T cells were cocultured with MDMs for 3 hr, T cells washed off, and MDMs cultured ± AZT for the times shown prior to lysis and qPCR for HIV-1 *pol* product normalized to *β-globin* copy number. Data represent mean relative HIV-1 vDNA copies from four independent donors + SEM. ^∗^p < 0.05, as determined by Kruskal-Wallis one-way ANOVA with Dunn’s multiple comparison post hoc test, normalized to T = 0 + AZT. (B) HIV-1_BaL_-infected primary CD4^+^ T cells directly cocultured with autologous MDM (CTC) or separated by virus-permeable transwell (TW) membranes for the times shown were washed, further cultured, and supernatant Gag assayed. Results are means of experimental quadruplicates+ SEM from an experiment representative of three independent experiments each with an independent donor. See Figures S2A–S2C. (C) HIV-1_BaL-GFP_^+^ primary T cells were cocultured for 6 hr with autologous MDMs; extensively washed; and MDMs were cultured for 3 days, fixed, permeabilized, and prepared for confocal microscopy. Each image represents a single optical *x-y* section, cells labeled for CD3 (red) and nuclei (blue). Scale bars = 20 μm. Top panel, MDM exposed to cell-free HIV-1_BaL-GFP_ through 3 μm transwell membranes; middle panel: MDM + AZT prior to and during coculture with HIV-1_BaL-GFP_^+^ T cells; bottom panel: MDMs directly cocultured with HIV-1_BaL-GFP_^+^ T cells. (D) Quantification from images represented in (C). MDMs under each condition were quantified for GFP expression, and data was expressed as means of + SEM of n = 500 MDMs in five randomly selected fields from four independent donors. ^∗^p < 0.05 Kruskal-Wallis one-way ANOVA with Dunn’s multiple comparison post hoc test. (E) Jasplakinolide inhibits HIV-1^+^ T-cell-mediated, but not cell-free, infection of MDMs. Jasplakinolide (Jasp, 5 μM) was added to MDMs for 1 hr prior to washing and direct coculture with HIV-1_Bal_^+^ T cells (CTC, red) or their supernatants (CF, black). Infection was measured by supernatant Gag levels 3 days post-coculture. Bars = mean inhibition of infection (%) + SEM for four independent donors, each in triplicate. ^∗∗∗^p < 0.001 by one-sample t test, comparing each condition to a hypothetical value of 0 with Sidak’s multiple comparison post hoc test. See Figure S2. (F) HIV-1 entry inhibitors block MDM infection. MDMs were cocultured with autologous HIV-1_BaL_^+^ T cells for 3 hr, washed extensively to remove unattached T cells, and cultured with entry inhibitors for 14 days with Gag release assayed every 2 days. Bars = AUC analysis normalized to no drug controls and expressed as inhibition of infection (%) + SEM for four donors, each in triplicate. ^∗^p < 0.05, ^∗∗^p < 0.01 by one-sample t test, comparing each condition to a hypothetical value of 0 with Sidak’s multiple comparison post hoc test. See Figure S3. (G) MDMs derived from a Δ32 CCR5 homozygous donor are resistant to infection by HIV-1^+^ T capture. MDMs from five CCR5 WT donors or a Δ32 CCR5 homozygote were cocultured with heterologous HIV-1_BaL-Luc_^+^ T cells for 6 hr, washed, and cultured for a further 3 days prior to lysis and measurement of luciferase activity. Bars equal means of quadruplicates + SEM. ^∗^p < 0.05 Mann Whitney U. See Figure S5A. (H and I) MDM infection by HIV-1^+^ T cell capture results in release of infectious virus. MDMs from four independent donors were cocultured with HIV-1_BaL-Luc_^+^ T cells directly or across a transwell (TW) for 6 hr prior to washing and further culture ± AZT for 3 days. (H) Supernatants from MDMs without AZT were titrated onto TZM-bl indicator cells and infectious units/ml determined. (I) MDMs washed to remove free virus were cocultured with autologous CD4^+^ T cells ± AZT for 24 hr prior to collection of T cells. T cells were washed and maintained ± AZT for 3 days prior to lysis and luciferase assay. Bars = means + SEM, ^∗^p < 0.05 by paired Students t test.

Potential outcomes of HIV-1^+^ T cell uptake by MDM include phagolysosomal elimination without MDM infection or viral spread from T cells to infect MDM. To interrogate this we prepared and cocultured MDM with HIV-1^+^ T cells (Figure 1B), washed and maintained cultures up to 72 hr ± AZT, and measured vDNA content. The signal rose by >10-fold from baseline without AZT demonstrating active HIV-1 replication, whereas with AZT it decreased by ∼3-fold, implying vDNA degradation (Figure 3A). We compared infection kinetics of MDM directly exposed to HIV-1^+^ T cells or across transwells and assayed released viral p24 Gag representing productive infection. Direct coculture of HIV-1^+^ T cells with MDM for 3 hr or longer yielded robust and prolonged Gag release (Figure 3B). By contrast, 3 hr exposure of MDM to HIV-1^+^ T cells across transwells did not result in a spreading infection, and 6 and 12 hr transwell exposure resulted in delayed Gag release with substantially lower peak values.

Although these data imply MDM infection, a proportion of Gag release might result from virus production by MDM-captured HIV-1^+^ T cells. We therefore investigated whether MDM became infected in the absence of residual T cells. CD4^+^ T cells were infected with HIV-1_NL4.3-eGFP-BaL_, cocultured with MDM for 3 days to approximate a single-cycle MDM infection, washed, and imaged. We observed ∼5% MDM with strong cytoplasmic GFP expression in the absence of detectable T cells (Figures 3C and 3D) that was eliminated by AZT treatment, demonstrating direct MDM infection, whereas cell-free infection yielded an insignificant GFP^+^ MDM signal. To further investigate the link between HIV-1^+^ T cell capture and MDM infection, we maintained cocultures for 3 days following uptake inhibition experiments and measured Gag release. Jasplakinolide, which reduced HIV-1^+^ T cell uptake (Figure 2B), strongly (p < 0.01) inhibited T-cell-mediated MDM infection but weakly (p > 0.05) reduced cell-free infection (Figure 3E). We tested for inhibition of MDM infection by extended culture of MDMs exposed to HIV-1^+^ T cells, in the continued presence of entry inhibitors. All inhibitors reduced MDM infection (Figure 3F), demonstrating that although these reagents do not prevent HIV-1^+^ T cell capture by MDM (Figure 2B), they act at a subsequent step to block viral entry into MDM. Therefore MDM infection through this route is dependent on Env-receptor interactions. Further confirmation of MDM infection by HIV-1^+^ T cell capture was obtained using MDMs derived from an individual homozygous for the CCR5 Δ32 mutation, which are resistant to R5 HIV-1 infection (Bol et al., 2009). These MDMs were confirmed CCR5 null compared to cells from WT individuals (Figure S5A). Heterologous CCR5^+^ primary CD4^+^ T cells infected with HIV-1_BaL-Luc_ were directly cocultured with, or exposed across transwells to, ΔCCR5 or WT donor MDM for 6 hr, washed, cocultured for 3 days, and assayed for luciferase. Figure 3G shows that the ΔCCR5 MDMs produced weak signals not significantly above baseline, probably reflecting residual T-cell-associated HIV-1, whereas the four WT donor MDM signals were ∼10-fold higher for both direct coculture and cell-free infection, demonstrating MDM infection.

To investigate the possibility that HIV-1 transfer from T cells to MDMs yields an abortive infection at a late stage of viral replication, we cocultured MDM with HIV-1^+^ T cells directly or across transwells for 6 hr, washed, cultured for 3 days, and assayed supernatant infectivity on TZM-bl cells. Significantly more infectious virus (p < 0.05) was released from MDM directly exposed to infected T cells than exposed to cell-free virus (Figure 3H). We also added autologous CD4^+^ T cells ± AZT to these MDM to act as indicators of onward cell-to-cell HIV-1 spread from MDMs to T cells. This revealed efficient HIV-1 infection of the reporter T cells that was greater in magnitude from MDMs initially cocultured with infected CD4^+^ T cells than infected across transwells, and infection was abolished by AZT (Figure 3I). Taken together, these data demonstrate that exposure of MDMs to HIV-1-infected CD4^+^ T cells leads to robust and productive MDM infection that is greater in magnitude, at all time points and under all conditions tested, than exposure to the cell-free viral counterpart.

### HIV-1^+^ T-Cell-Mediated Infection of MDM and Viral Tropism

**Figure 4:**
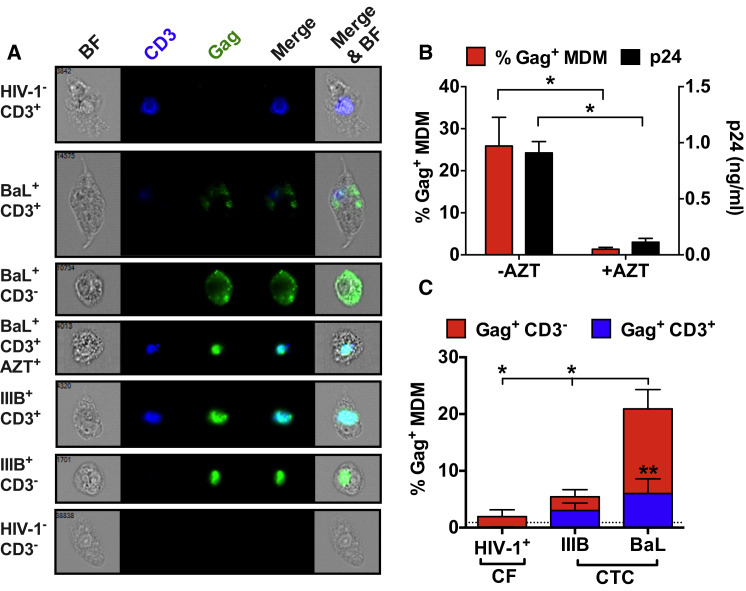
HIV-1^+^ T-Cell-Mediated Infection of MDMs and Viral Tropism (A) NM-tropic HIV-1 does not establish significant infection in MDMs. ImageStream images selected from 5 × 10^4^ total acquired events gated for MDMs. HIV-1_BaL_ supernatant virus or HIV-1_BaL_- or HIV-1_IIIB_-infected primary T cells cocultured for 6 hr with autologous MDMs were washed free of unattached T cells, cultured for 6 days, fixed, and processed for ImageStream analysis. Cells were labeled for CD3 (blue) and Gag (green), brightfield = BF. (B) Quantification of data from assay as in (A) showing % MDM containing Gag and corresponding supernatant p24 Gag levels after coculture with HIV-1_BaL_^+^ T cells ± 1 hr AZT pretreatment. Where appropriate AZT was maintained in coculture and on MDMs post-coculture for 6 days. Data are from two independent experiments with four independent donors, p24 analyzed in triplicate. Bars = mean % Gag^+^ MDM or p24 (ng/ml) + SEM *^∗^*p < 0.05 by Mann-Whitney U comparing CTC to AZT for each measure. (C) Summary of data quantified from images such as those in (A). Bars represent % Gag^+^ MDM + SEM from four independent donors, separated into CD3-associated (blue) and non-CD3-associated (red). ^∗^p < 0.05 by one-way ANOVA with Bonferroni’s multiple comparison post hoc test comparing total % Gag^+^ MDM between cell-to-cell HIV-1_BaL_ and HIV-1_IIIB_ or HIV-1_BaL_ cell-to cell to HIV-1_BaL_ cell-free. *^∗^*^∗^p < 0.01 by Student’s t test comparing the proportion of CD3-associated (blue) to non-CD3-associated (red) Gag. See Figures S5B–S5D.

We hypothesized that the efficiency of MDM infection by HIV-1^+^ CD4^+^ T cell uptake might influence viral tropism, and we used ImageStream to investigate the fate of macrophage (M)-tropic and nonmacrophage (NM)-tropic HIV-1-infected T cells associated with MDM. We confirmed that MDM cocultured with M-tropic HIV-1_BaL_^+^ T cells for 6 days were productively infected: ∼25% of MDMs expressed cytoplasmic Gag and released free Gag p24, and these signals were eliminated by AZT treatment (Figure 4A and 4B). T cells infected with NM-tropic X4 virus HIV-1_IIIB_ were taken up by MDMs (Figure 4A), as anticipated from the results in Figure 1A and Movie S1. However, the majority of the HIV-1_IIIB_ Gag signal within the MDM was associated with CD3, suggesting internalized infected T cells, and the remainder with CD3-negative vesicular compartments possibly representing degraded T cells that had lost CD3 expression (Figures 4A and 4C). By contrast, the Gag signal in MDMs cocultured with HIV-1_BaL_^+^ T cells was predominantly (∼75%) not associated with a CD3 signal (Figures 4A and 4C), implying infection of ∼15% MDMs in the absence of residual T cells or debris. However, this analysis only detects CD3 and Gag signal within the same MDM and does not differentiate MDMs that contain a CD3 signal with a nonassociated cytoplasmic Gag signal, which would imply MDM infection in the presence of residual T cell material. To probe this, we established a brightfield mask to define the MDM and then excluded CD3^+^ regions from this mask using a stringent CD3 mask. This analysis revealed that for HIV-1_BaL,_ ∼22% MDMs were Gag^+^ within which ∼18% contained non-CD3-associated cytoplasmic Gag. For HIV-1_IIIB_ ∼6% MDMs were Gag^+^ within which ∼4% was non-CD3-associated, a signal not significantly above baseline (Figure S5B).

The CD3-associated HIV-1 Gag signal within MDMs might represent a small population of viable T cells able to propagate infection, or CD3^+^ debris from degraded HIV-1^+^ T cells. To interrogate this we created a gate to report only CD3 signal representing an intact T cell, based on shape and size (round, >100 pixels in area, equivalent to 25 μm^2^, approximately the area of a T cell), thereby excluding CD3^+^ debris (Figure S5C). We found that ∼2%–3% of all MDMs contained intact T cells, corresponding to ∼6% of both HIV-1_BaL_ and HIV-1_IIIB_-infected MDMs. Furthermore, when these internalized intact T cells were analyzed for infection, the majority (55%–70%) were Gag^+^ (Figure S5D). These data reveal that a small proportion of HIV-1-infected MDMs contain potentially viable HIV-1^+^ T cells several days postuptake but that infection of MDM, as reported by a significant proportion of cytoplasmic Gag within MDM, only takes place with M-tropic HIV-1.

### T Cell Capture Facilitates T/F Virus Infection of MDM

**Figure 5:**
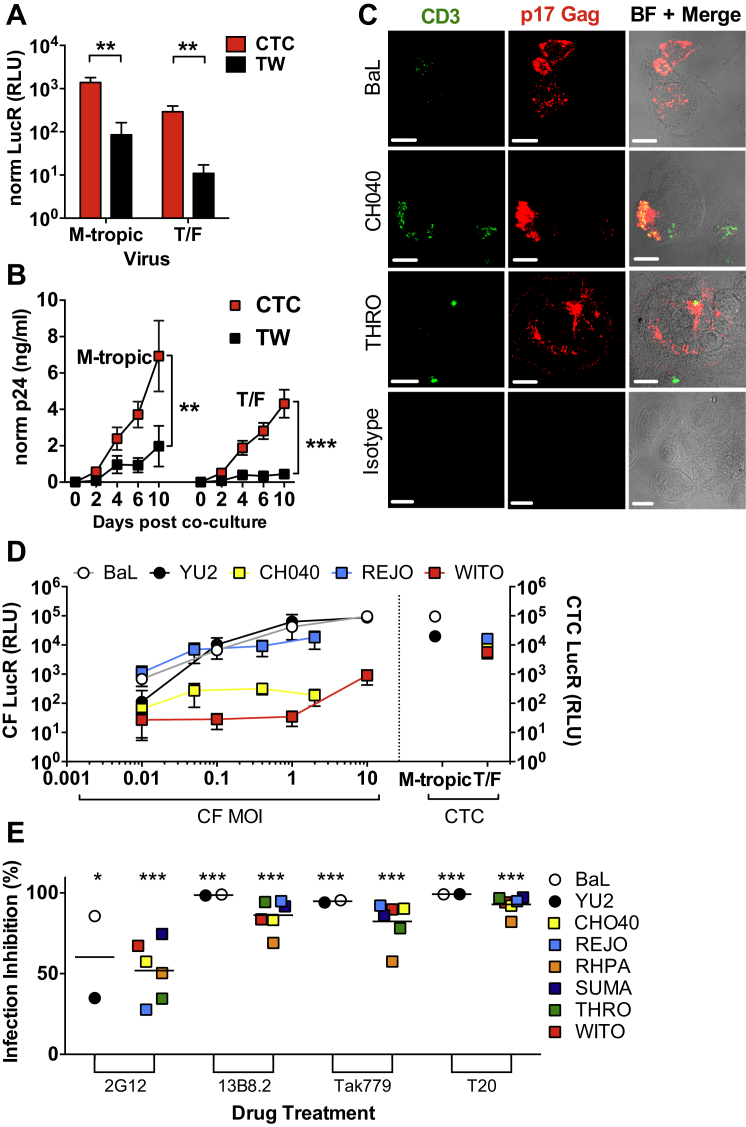
T Cell Capture Facilitates T/F Virus Infection of MDM (A) Primary T cells infected individually with IMCs expressing two M-tropic R5 Envs (BaL, YU2) or 6 T/F Envs (CH040, THRO, RHPA, WITO, REJO, and SUMA) (Ochsenbauer et al., 2012) were cultured for 6 hr directly (CTC) or across transwells (TW) with autologous MDMs. Following washing, MDMs were cultured for 6 days, lysed, and luciferase assayed. Results are mean signal + SEM for each individual IMC data set pooled into M-tropic or T/F groups from four independent donors, and values are normalized to % Gag^+^ T cells prior to coculture. ^∗∗^p < 0.01 Kruskal-Wallis ANOVA plus Dunn’s multiple comparison post hoc test. See Figure S5E. (B) MDMs were cocultured with HIV-1^+^ T cells as in (A), cultured for 10 days, and supernatant Gag measured and normalized as above. Boxes represent mean normalized Gag ± SEM of four independent donor infections in two independent experiments, each analyzed in triplicate. AUC analysis ^∗∗^p < 0.01; ^∗∗∗^p < 0.001. (C) MDM cocultured with primary autologous CD4^+^ T cells infected with the IMCs expressing Env from HIV-1_BaL_, HIV-1_CH040_, or HIV-1_THRO_ for 7 days were washed to remove T cells cultured for a further 6 days; fixed; and labeled for confocal fluorescence microscopy with anti-CD3 (green) and Gag p17 (red) antibodies. BF = brightfield; scale bars = 20 μm. (D) MDMs were cocultured with T cells infected with the IMCs shown or infected with concentrated cell-free virus at the MOIs shown for 6 hr, cultured for a further 3 days, washed, lysed, and luciferase assayed. Results are mean signal ± SEM for four independent donors. Left panel is cell-free (CF) infection; right panel is cell-to-cell (CTC) infection. (E) MDMs were cocultured with IMC-infected T cells for 3 hr with the inhibitors shown, washed, and cultured for a further 3 days in the continued presence of the inhibitors. MDM, were washed, lysed, and assayed for luciferase activity. Individual datum points = the mean of three to four independent donors from two independent experiments for each virus tested. Lines represent the overall mean for each group (M-tropic or T/F). ^∗^p < 0.05; ^∗∗∗^p < 0.001 by one-sample t test comparing means to a hypothetical value of 0 (no inhibition) with Sidak’s multiple comparison post hoc test.

Although HIV-1 transfer from infected T cells to MDMs does not appear to expand the tropism of NM-tropic HIV-1, it may influence infection efficiency of viruses weakly tropic for MDMs. We therefore investigated whether R5 T/F viruses that are predominantly T cell tropic by cell-free infection (Ochsenbauer et al., 2012, Salazar-Gonzalez et al., 2009) might infect MDMs more efficiently via HIV-1^+^ T cell uptake. MDMs were cocultured directly, or across transwells, with CD4^+^ T cells infected independently with six different T/F viruses or two chronic M-tropic luciferase-encoding IMCs (Figure S5E) as before and assayed for infection. Cell-free infection by M-tropic HIV-1 IMCs gave robust luciferase signals at day 6 (Figure 5A) and Gag release over 10 days (Figure 5B). By contrast, cell-free T/F virus MDM infection yielded ∼10-fold lower levels of luciferase than HIV-1_BaL_ and HIV-1_YU2_ (Figure 5A) and insignificant Gag release compared to baseline over 10 days (Figure 5B), consistent with previous reports (Ochsenbauer et al., 2012, Salazar-Gonzalez et al., 2009). However, direct coculture of T/F virus-infected T cells with MDMs yielded ∼30-fold greater day 6 luciferase and significantly (p < 0.001) greater Gag release than cell-free infection (Figure 5B). T/F virus infection of MDMs was confirmed by imaging MDMs at day 6 postcoculture, demonstrating extensive cytoplasmic Gag in the absence of intact T cells, although residual scattered CD3^+^ T cell debris was evident in some MDMs (Figure 5C). The enhanced ability of T/F viruses to infect MDMs via cell-to-cell spread from infected T cells might be explained by an increased multiplicity of infection (MOI) imparted by this mode of spread. To test this we exposed MDM to increasing titers of cell-free M-tropic or T/F IMCs and evaluated MDM infection after 3 days. M-tropic HIV-1_BaL-luc_ and HIV-1_YU2-luc_ gave robust luciferase signals that increased with input cell-free virus to >10^5^ RLU at MOI = 10, overlapping that obtained by capture of HIV-1^+^ T cells (Figure 5D). A similar range of signals was also observed for the T/F virus REJO up to the maximum obtainable MOI of 2. Thus, the efficiency of cell-to-cell infection is recapitulated for these viruses by high-MOI, cell-free infection. By contrast, despite similar input cell-free MOI maxima of 10 for WITO and 2 for CH040, infection levels remained low, giving maximum luciferase values 5- to 50-fold below that obtained by the cell-to-cell route of MDM infection (Figure 5D). Increasing the MOI of cell-free T/F virus inocula therefore increased MDM infection in a virus-strain-dependent manner. Since T/F viruses display reduced tropism for MDMs compared to chronic M-tropic viruses and may have different sensitivity to inhibitors of CD4- and CCR5-gp120 binding (Chikere et al., 2014, Ochsenbauer et al., 2012, Parker et al., 2013), we compared T/F virus inhibition by the same entry inhibitors used against HIV-1_Bal_ (Figure 3F). MDMs were cocultured with autologous T cells infected with the IMC panel, washed, and cultured in the continued presence of the inhibitors for 6 days. All inhibitors strongly inhibited MDM infection by all viruses with the exception of 2G12, which only weakly inhibited neutralization-resistant strain HIV-1_YU2_ and some T/F viruses (Figure 5E). These data confirm that MDM infection via capture of M-tropic and T/F virus-infected T cells is equivalently sensitive to entry inhibition.

## Discussion

Our results reveal a mechanism of efficient HIV-1 cell-to-cell spread driven by MDM capture of HIV-1^+^ T cells. The finding that macrophage infection may be initiated in this way has implications for infection in vivo. Macrophage recognition and uptake of HIV-1-infected healthy and dying T cells might take place at all times during natural infection, and both live and dead/dying T cells are potent carriers of infectivity (Sealy et al., 2009). This mode of viral spread could occur shortly after transmission when T/F viruses replicate locally in Lamina Propria CD4^+^ T cells and later when massive infection and death of CD4^+^ T cells in secondary lymphoid tissues (Li et al., 2005, Mattapallil et al., 2005) might drive substantial macrophage uptake of HIV-1^+^ T cells. Conversely, the finding that NM-tropic viral Gag may persist within T cells engulfed by macrophages may impact upon interpretation of data from macrophages obtained ex vivo from HIV- or SIV-infected hosts. Detection of vDNA or viral proteins within phagocytes including macrophages and monocytes may not necessarily represent their infection but may indicate uptake of infected immune cells or their debris.

We show that HIV-1-infected T cells are captured by MDMs using interactions apparently distinct from those mediating conventional VS formation (Sattentau, 2008) but have yet to define the uptake recognition signals. Since immunodeficiency virus-infected T cells die in vitro and in vivo (Li et al., 2005, Mattapallil et al., 2005), we explored macrophage recognition of this process by attempting to block death receptor-ligand interactions using published inhibitors. However, none significantly reduced HIV-1^+^ T cell capture. This might reflect the highly redundant nature of these interactions (Devitt and Marshall, 2011, Poon et al., 2010), or that the receptor-ligand interactions implicated in this process are as yet undefined, or that the pathway of macrophage recognition of dead/dying cells is distinct from that used for HIV-1^+^ T cell recognition. We have not explored the cell biology of HIV-1 infection of macrophages following capture of infected T cells here. Uptake of HIV-1^+^ T cells by macrophages leading to T cell degradation in a maturing lysosomal environment may indeed occur for a proportion of engulfed T cells. However, it is evident from our data that some virus escapes from infected T cells prior to degradation, leading to robust macrophage infection. Whether this takes place during HIV-1^+^ T cell capture at the plasma membrane or later from within the uptake compartment remains to be determined. Regardless of the membrane with which virus fuses, entry via this mode of infection is dependent on the expression of CD4 and CCR5, as demonstrated by our inhibitor analyses. A somewhat surprising finding was that a small percentage of engulfed T cells remained intact within macrophages for up to 6 days and possibly longer. Although it is unclear whether any of these cells were viable, it appears that they were taken up into a nondegradative compartment within which they persisted for extended periods.

The magnitude of MDM infection achieved by capture of T cells infected by the M-tropic viruses and the T/F virus REJO, which we have previously described as having a stronger macrophage tropism than most T/F viruses (Ochsenbauer et al., 2012), was recapitulated by a high cell-free MOI infection. Thus, for these viruses, the efficiency of this mode of macrophage infection is probably determined solely by quantitative aspects of virus transfer from the infected T cell. However, even at high MOI, two other T/F viruses (WITO and CH040) did not achieve a level of infection equivalent to that of cell-to-cell infection, an observation consistent with the particularly weak MDM tropism of these viruses (Ochsenbauer et al., 2012, Salazar-Gonzalez et al., 2009). Increasing the cell-free MOI to >10 may overcome entry restrictions to WITO and CH040, giving an equivalent infection to the cell-to-cell route. However, since T/F viruses appear to have a reduced affinity for CD4 and altered interaction with CCR5 compared to M-tropic viruses (Ochsenbauer et al., 2012, Parker et al., 2013), a further possibility is that capture of HIV-1-infected T cells by macrophages may drive clustering of viral receptors at the interface, facilitating virus-receptor engagement and entry as reported for VS formation between HIV-1-infected and uninfected T cells (Jolly et al., 2004).

The idea that a pathogen may disseminate from one infected cell type to another by phagocyte engulfment is not new and has been termed a “Trojan horse” strategy with respect to *Leishmania major* parasite dissemination within an infected host (Ritter et al., 2009). In this model, neutrophils that engulf *L. major* harbor the parasites in an infectious form, ultimately dying and passing them onto their principal host cell, the macrophage (Peters et al., 2008). Similarly, macrophages that have engulfed *Mycobacterium tuberculosis* bacilli undergo apoptosis and are subsequently phagocytosed by newly recruited macrophages, efficiently spreading the infection (Davis and Ramakrishnan, 2009). Finally, *Listeria monocytogenes*-induced PS exposure on infected cells mimics apoptotic signals that enhance cell-to-cell spread of this bacterium to macrophages (Czuczman et al., 2014). Our observation of the capture of HIV-1-infected T cells by macrophages extends this phenomenon to viruses and may have important implications for spread of viruses to phagocytic cells within infected hosts.

## Experimental Procedures

### Cells and Virus

MDM were generated and maintained as detailed in Groot et al. (2008), except that monocytes were negatively selected from donor peripheral blood mononuclear cells using monocyte isolation kit-II (Miltenyi Biotec). Autologous CD4^+^ T cells were negatively selected from PHA/IL-2-stimulated peripheral blood mononuclear cells (CD4 isolation kit, Miltenyi Biotec) and infected with the following viruses: WT HIV-1_BaL_ or HIV-1_IIIB_ (NIBSC Centre for AIDS Reagents, CFAR), 5–50 ng CA-p24 per 1 × 10^7^ CD4^+^ T cells for 7 days in complete RPMI 1640/10% fetal calf serum/10 U/ml IL-2 (CFAR). Replication-competent IMC stably expressing *Renilla reniformis* luciferase in an isogenic NL4.3 backbone (NL-LucR.T2A) encoding diverse *env* ectodomains in *cis* (Ochsenbauer et al., 2012) were prepared by 293T transfection and titered on TZM-bl cells. Envs were R5 M tropic (BaL, YU2), R5 T/F (RHPA, THRO, WITO, REJO, SUMA and CH040), and X4 NL4.3 NM tropic (Ochsenbauer et al., 2012). T cells were 10%–20% Gag^+^ on the day of use as determined by flow cytometry (FACSCalibur, BD Biosciences). For live-cell imaging experiments, Jurkat-Tat-R5 or primary CD4^+^ T cells were infected with the X4 HIV-1_NL4.3-GFP_ or mCherry IMC expressing R5 T/F Env CH077 (NL4.3-RFP (HIV-1_CH077/mCherry_) by magnetofection (Sacha and Watkins, 2010) to achieve >15% GFP^+^ or mCherry^+^ cells at 2 days postinfection.

### Viral Transmission Experiments

T cell to MDM HIV-1 transmission was by direct coculture or across transwell membranes (3.0 μm, Costar) or cell-free supernatant added directly to target cells. Within-donor variability in T cell infection levels between IMCs in MDM infection readouts was normalized in Figures 5A and 5B to starting T cell infection levels. In some experiments, MDMs were incubated with AZT (5 μM, CFAR) for up to 12 hr pre-coculture and maintained in the medium. For qPCR assay of vDNA, total DNA was extracted with the DNeasy blood and tissue kit (QIAGEN) and amplified using HIV-1 *pol* and *β-globin* primers (Martin et al., 2010), and results were expressed as *pol*:β-globin to normalize to cell number. For luciferase readout, cell lysates were prepared (Glo-Lysis buffer, Promega) and 50 μl lysate mixed with 50 μl Ren-Glo luciferase solution (Promega) at RT and activity (relative light units, RLU) measured after 10 min. For GFP-reporting of MDM infection, PHA/IL-2-activated CD4^+^ T cells were synchronously magnetofected or mock infected for 48 hr with HIV-1_BaL-GFP_. T cells were washed, added to autologous MDM 1:1, and cocultured for 6 hr. Following coculture, free or loosely attached T cells were washed off with 10 mM EDTA, MDMs were cultured, and productive infection was analyzed at day 3 post-coculture by fixing and confocal microscopy. For VSVg-mediated HIV-1 transduction, PHA/IL-2-activated CD4^+^ T cells were synchronously magnetofected for 48 hr with HIV-1_BaL_ or VSV-G pseudotyped HIV-1_BaLΔEnv_ (CFAR, NIBSC) or mock infected. Infected CD4^+^ T cells were added to autologous MDM (1:1) and cocultured for 3 hr. MDMs were washed before lifting in 5 mM EDTA/12 mM lidocaine, fixation, permeabilization, staining, and analysis by flow cytometry. For p24 ELISA, supernatants were removed, centrifuged, and inactivated with 0.5% empigen/56°C (Groot et al., 2008).

### Inhibitors of Viral Entry, HIV-1^+^ T Cell Uptake, and Cellular Function

Inhibitors of MDM uptake were jasplakinolide (5 μM, Molecular Probes) and 5-N-ethyl-N-isopropyl amiloride (EIPA, 50 μM, Sigma-Aldrich). Inhibitors of HIV-1 Env-receptor interactions were 13B.8.2, CD4-specific gp120 blocking mAb (10 μg/ml Beckman-Coulter); soluble CD4 (20 μg/ml, IAVI); Tak-779 (500 nM, CFAR); 2G12 (10 μg/ml, IAVI); and T20 (7.5 μg/ml, CFAR). Relevant isotype controls were used at matched concentrations. MDM Fc receptors were blocked in 10 μg/ml pooled human IgG at 37°C for 1 hr. Inhibitors were preincubated with MDMs and/or T cells at 37°C and maintained during coculture (HIV-1 Env receptor inhibitors) or washed out after preincubation with MDMs (MDM uptake inhibitors). MDMs were pretreated with 13B8.2, T20, Tak-779, EIPA, or jasplakinolide. HIV-1-infected T cells were pretreated with sCD4, 2G12, or T20.

### Confocal Microscopy

Performed as described previously in Groot et al. (2008). Cells were fixed in 4% paraformaldehyde (PFA) for 1 hr, quenched with 50 mM NH_4_Cl in PBS for 20 min, nuclei stained with 1 μg/ml Hoechst in PBS, and cells permeabilized in wash buffer (WB, 0.1% saponin/0.5% BSA in PBS + 5% pooled human and goat serum). Samples were stained for CD3 (UCHT1-IgG1, BD Biosciences or UCHT1-IgG2a, P. Beverley), mouse anti-HIV-1 Gag p17 (CFAR, 4C9) with anti-mouse Alexa 488/647-IgG1 or -IgG2a (Invitrogen). Alternatively, directly conjugated mouse anti-HIV-1 Gag KC57-FITC (Beckman-Coulter) and phalloidin-TRITC (Sigma) were used. Coverslips were mounted in ProLongGold Antifade (Invitrogen) and analyzed using an Olympus FV1000. Images were acquired with a 60× oil-immersion objective and processed in Olympus Fluoview v2.0b.

### Multispectral Flow Cytometry (ImageStream)

MDM were differentiated for 7 to 10 days. Autologous HIV-1_BaL_-infected CD4^+^ T cells were unlabeled or labeled with anti-PS-FITC (Millipore), Live/Dead (LD)-Red (Invitrogen). In PS prelabel experiments, cells were postlabeled with CD3-PerCP Cy5.5 (BD Biosciences), active Caspase3-APC (BD Biosciences), and Gag (p17, p24, and p55) KC57-PE (Beckman Coulter). For LD experiments or experiments with no T cell prelabel, cells were labeled with CD3-APC (BD Biosciences), Caspase3-PE (BD Biosciences), and Gag KC57-FITC (Beckman Coulter). MDMs and T cells were cocultured (1:1) for 3 hr, washed extensively with PBS/EDTA (10 mM), and lifted with cold PBS/EDTA (5 mM)/lidocaine (12 mM). Cells were fixed in 4% PFA and permeabilized. T cell phenotypes (Figures S1A–S1H) and T cell phenotypes associated with MDMs (Figures S1I–S1N) were determined. From this population, 10^3^ MDMs per donor were assessed manually, and internalized T cell phenotypes were scored. The proportions of each T cell phenotype associated with MDMs were determined using the total number of T cells counted per 10^3^ MDMs as the denominator. Uptake index equals proportion of T cell population phenotypes pre-coculture/MDM-associated T cell phenotypes. For long-term infection experiments, HIV-1_BaL_^+^ or HIV-1_IIIB_^+^ CD4^+^ T cells (1:1) or 6 hr HIV-1_BaL_^+^ T cell supernatants were cocultured with MDM for 6 hr prior to extensive washing. MDMs were maintained for 3 to 6 days, lifted, fixed (as above), and stained with CD3-APC and Gag KC57-FITC. Samples were processed using ImageStream 100 or ImageStream^X^ and analyzed with IDEAS 4.0 or 5.0 software (Amnis).

### Flow Cytometry

Autologous HIV-1_BaL_^+^ CD4^+^ T cells were labeled with PS-FITC, LD-Red, or unstained prior to coculture and prepared as for ImageStream. Antibodies used were as follows: CD14-APC (BioLegend), CD14-FITC (BD Biosciences), CD3-FITC, CD3-APC, KC57-FITC/PE, CD4-APC, or CCR5-FITC, together with appropriate fluorochrome-conjugated isotype controls. Cells were gated according to scatter and staining for CD14 (MDM) or CD3 (T cell). Samples were collected on a FACSCalibur (BD) and analyzed with FlowJo.

### Statistical Analysis

All analyses were performed with Graphpad Prism V5 or V6, and data were initially interrogated for normal distribution. For group-wise comparisons, one-way ANOVA with Bonferonni post hoc test (parametric), Kruskal-Wallis one-way ANOVA with Dunn’s post hoc test (nonparametric), or two-way ANOVA with Bonferonni post hoc test (parametric) were used to account for multiple comparisons. Pairwise comparisons were done with Student’s t test or Mann-Whitney U. When comparing to a hypothetical mean, one-sampled t tests with Sidak’s post hoc test correction were used. All tests were two-tailed with alpha < 0.05 considered significant.

## Author Contributions

Q.J.S. and F.G. conceived the study; Q.J.S., F.G., A.E.B., R.A.R., and C.J.A.D. conceived, designed, and carried out experimental approaches; and Q.J.S., A.E.B., R.A.R., and C.J.A.D. wrote the paper. F.G., A.E.B., R.A.R., C.J.A.D., M.D.M., and C.W. performed, analyzed, and interpreted the experiments, and C.O., J.C.K., J.L.P., A.F., and D.E.K. supplied novel reagents. All authors have read, edited, and approved the final manuscript.

The authors declare no competing financial interests.

## Supplemental Information

Document S1. Figures S1–S5 and Supplemental Experimental Procedures

Movie S1. MDM Uptake of Three X4 HIV-1-Infected, GFP+ Jurkat T Cells

Movie S2. MDM Uptake of Three R5 T/F HIV-1-Infected mCherry+ Jurkat T Cells

Movie S3. MDM Uptake of an R5 T/F HIV-1-Infected mCherry+ Primary Autologous CD4+ T Cell

Document S2. Article plus Supplemental Information
