# Supplementary material for: Macrophage Infection via Selective Capture of HIV-1-Infected CD4+ T Cells
Source: Cell Host Microbe. 2014 Dec 10;16(6):711–21. doi: 10.1016/j.chom.2014.10.010 (PMC4271767; doi:10.1016/j.chom.2014.10.010)
Supplement: Document S1. Figures S1–S5 and Supplemental Experimental Procedures [file mmc1.pdf]

Cell Host & Microbe, Volume *16*

Supplemental Information

Macrophage Infection via Selective Capture of HIV-1-Infected $CD4^+$ T Cells

Amy E. Baxter, Rebecca A. Russell, Christopher J.A. Duncan, Michael D. Moore, Christian B. Willberg, Jose L. Pablos, Andrés Finzi, Daniel E. Kaufmann, Christina Ochsenbauer, John C. Kappes, Fedde Groot, and Quentin J. Sattentau

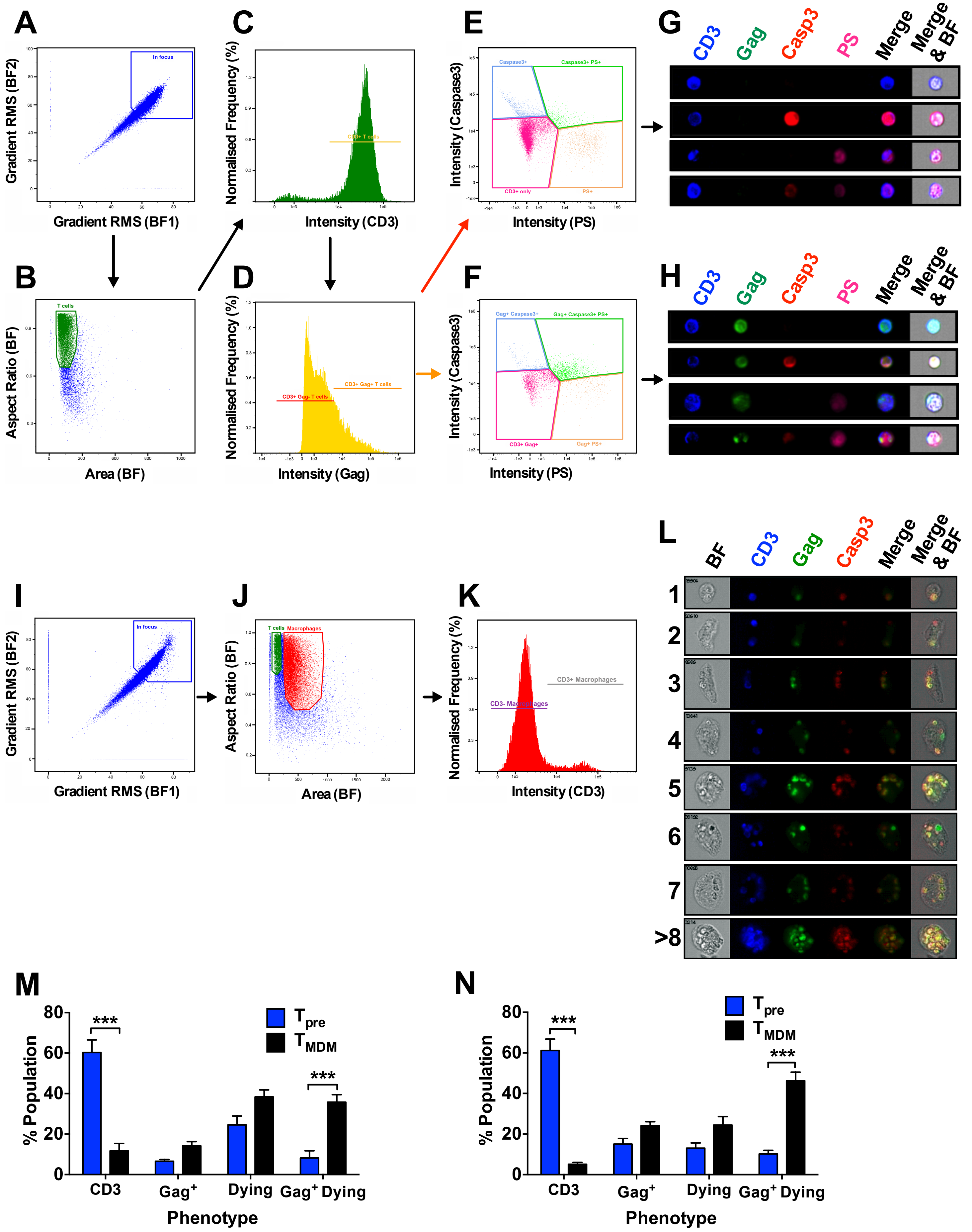

A
Gradient RMS (BF2)
Gradient RMS (BF1)
In focus
B
Aspect Ratio (BF)
Area (BF)
T cells
C
Normalised Frequency (%)
Intensity (CD3)
CD3+ T cells
D
Normalised Frequency (%)
Intensity (Gag)
CD3+ Gag- T cells
CD3+ Gag+ T cells
E
Intensity (Caspase3)
Intensity (PS)
Caspase3+
Caspase3+ PS+
CD3+ only
PS+
F
Intensity (Caspase3)
Intensity (PS)
Gag+ Caspase3+
Gag+ Caspase3+ PS+
CD3+ Gag+
Gag+ PS+
G
CD3
Gag
Casp3
PS
Merge
Merge & BF
H
CD3
Gag
Casp3
PS
Merge
Merge & BF
I
Gradient RMS (BF2)
Gradient RMS (BF1)
In focus
J
Aspect Ratio (BF)
Area (BF)
T cells
Macrophages
K
Normalised Frequency (%)
Intensity (CD3)
CD3- Macrophages
CD3+ Macrophages
L
BF
CD3
Gag
Casp3
Merge
Merge & BF
1
2
3
4
5
6
7
>8
M
% Population
Phenotype
CD3
Gag+
Dying
Gag+ Dying
Tpre
TMDM
***
N
% Population
Phenotype
CD3
Gag+
Dying
Gag+ Dying
Tpre
TMDM
***

Figure S1. ImageStream gating strategy for T cells and MDM with additional data analysis (associated with Figures 1C - E).

(A - H) ImageStream gating for T cells (associated with Figures 1C + 1E). HIV-1^{+} CD4 T cells prepared as described in methods were labeled for either LD (orange, Figure 1C + D) or PS (magenta, above), then fixed and labeled for CD3 (blue), Gag (green) and Caspase3 (Casp3, red). (A) T cell images were selected on the basis of focus from a total of 5 x 10^4 acquired, then (B) further selected on aspect ratio and area in the bright field channel. (C) CD3^{+} cells were selected based on CD3 intensity and (D) separated into infected (orange marker and arrow) and uninfected (red marker and arrow) based on Gag label. Each population was then analyzed for Caspase3 and LD or PS intensity (PS shown in E,F), and represented as single channel or merged images for Gag-negative (G) and Gag positive (H) cells. HIV-1^{+} CD4 (I - N) T cells prepared and labeled as described above were cocultured with autologous MDM for 3 hr, washed extensively to remove un-attached T cells, and MDM lifted, fixed, permeabilized and labeled for CD3, Gag and Caspase3 and analyzed by Imagestream™ (associated with Figures 1D - E). (I) MDM images were selected on the basis of focus from a total of 5 x 10^4 acquired then (J) further selected on area and aspect ratio in the bright field channel. (K) MDM associated with CD3^{+} T cells were selected on CD3 intensity and represented as single channel images or merged with or without bright field. (L) Representative images showing single or multiple T cells engulfed by MDM selected from 1000 images gated as above. (M, N) Unprocessed quantification of phenotypes of T cells stained for LD (M) or PS (N) in pre-coculture pool (blue bars) or post-coculture within MDM (black bars) (corresponding to Figure 1E). The healthy T cell population is significantly (***$p<0.001$) under-represented in MDM, whereas infected and dying or dead cells are significantly over-represented (***$p<0.001$). Two-way ANOVA with Sidak's multiple comparison post-test.

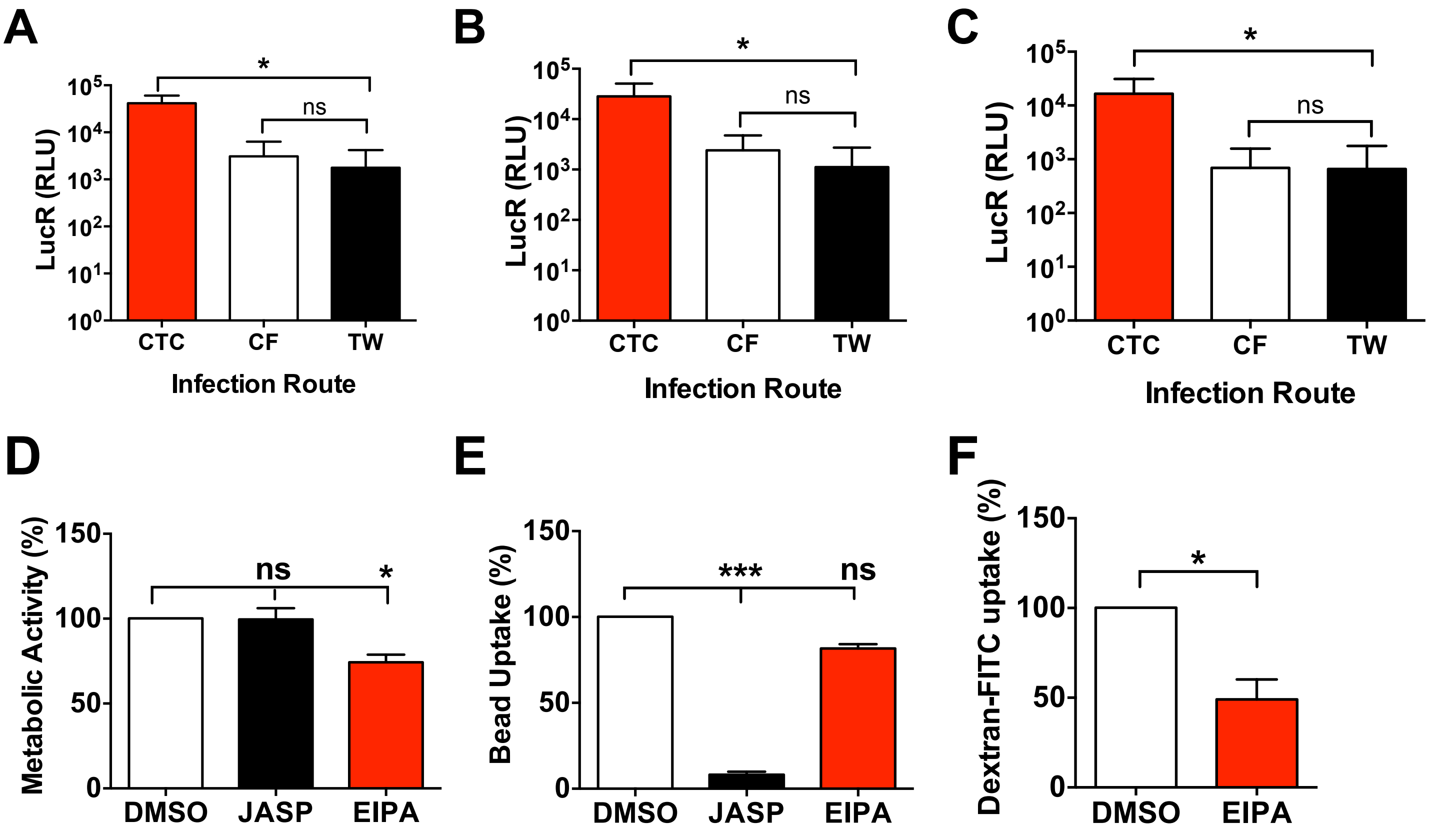

Figure S2. Macrophage infection route and drug toxicity controls (associated with Figures 2A, 2B, 3B, 3E, 3G - I, 4C, S4B, S4C, 5A, 5B + 5D, S5A +S5B).

(A, B + C) Comparison between different routes of cell-free infection of MDM with direct cell-to-cell spread of virus by coculture of HIV-1-infected T cells with MDM (associated with Figures 2A, 3B, 3E, 3G - I, 4C, 5A, 5B + 5D). MDM were cultured for 6 hours with supernatant from the same HIV-1$_{BaL\text{-}lucR}$-infected T cells (CF), or across a transwell (TW), or were directly cocultured with HIV-1$_{BaL\text{-}lucR}$-infected T cells (CTC) at ratios of (MDM:T cell): A) 1:2; B) 1:1 and C) 2:1. T cell numbers were the same for each condition (CF, CTC, TW). After washing off T cells or supernatants or removing transwells, MDM were cultured for 3 days, lyzed, luciferase activity assayed, and results expressed as relative light units (RLU). Bars represent means + SEM of a single experiment performed in triplicate with four independent donors. "ns" $p>0.05$, *$p<0.05$ by Kruskal-Wallis *t*-test with Dunn's multiple comparison post-test. (D, E + F) Toxicity and function of inhibitors of MDM uptake (associated with Figures 2B, 3E, S4B, S4C, S5A + S5B). (D) MDM were incubated with inhibitors of actin remodeling (Jasplakinolide , JASP, 5 µM), macropinocytosis (EIPA, 50 µM) or DMSO vehicle for 1 hr, washed and analyzed for metabolic activity by MTS assay. Bars represent mean metabolic activity expressed as % of DMSO-treated cells. *$p<0.05$ by one-sample t-test comparing each treatment to a hypothetical value of 100. (E) MDM were incubated with inhibitors as in (D) then washed and exposed to 6 µm alexa-488-conjugated latex microbeads (1:1) for 1.5 hr. MDM were washed, trypsinized, fixed and analyzed by flow cytometry. Bars represent mean bead uptake expressed as % of DMSO-treated cells in 2-4 independent experiments each assaying a different donor in triplicate. ***$p<0.001$ by one-sample *t*-test comparing each treatment to a hypothetical value of 100. (F) MDM were incubated with EIPA as in (D) then washed and incubated with 250 µg/mL of 150 kDa dextran-FITC for 30 mins at either 4°C or 37°C, then washed extensively, trypsinized, fixed and analyzed by flow cytometry. The dextran-FITC signal at 4°C was subtracted from that at 37°C to compensate for non-specific cell surface dextran-FITC binding. Bars represent mean dextran-FITC uptake + SEM expressed as % of DMSO-treated control cells in three independent experiments. *$p<0.05$ by one-sample *t*-test comparing EIPA to a hypothetical value of 100.

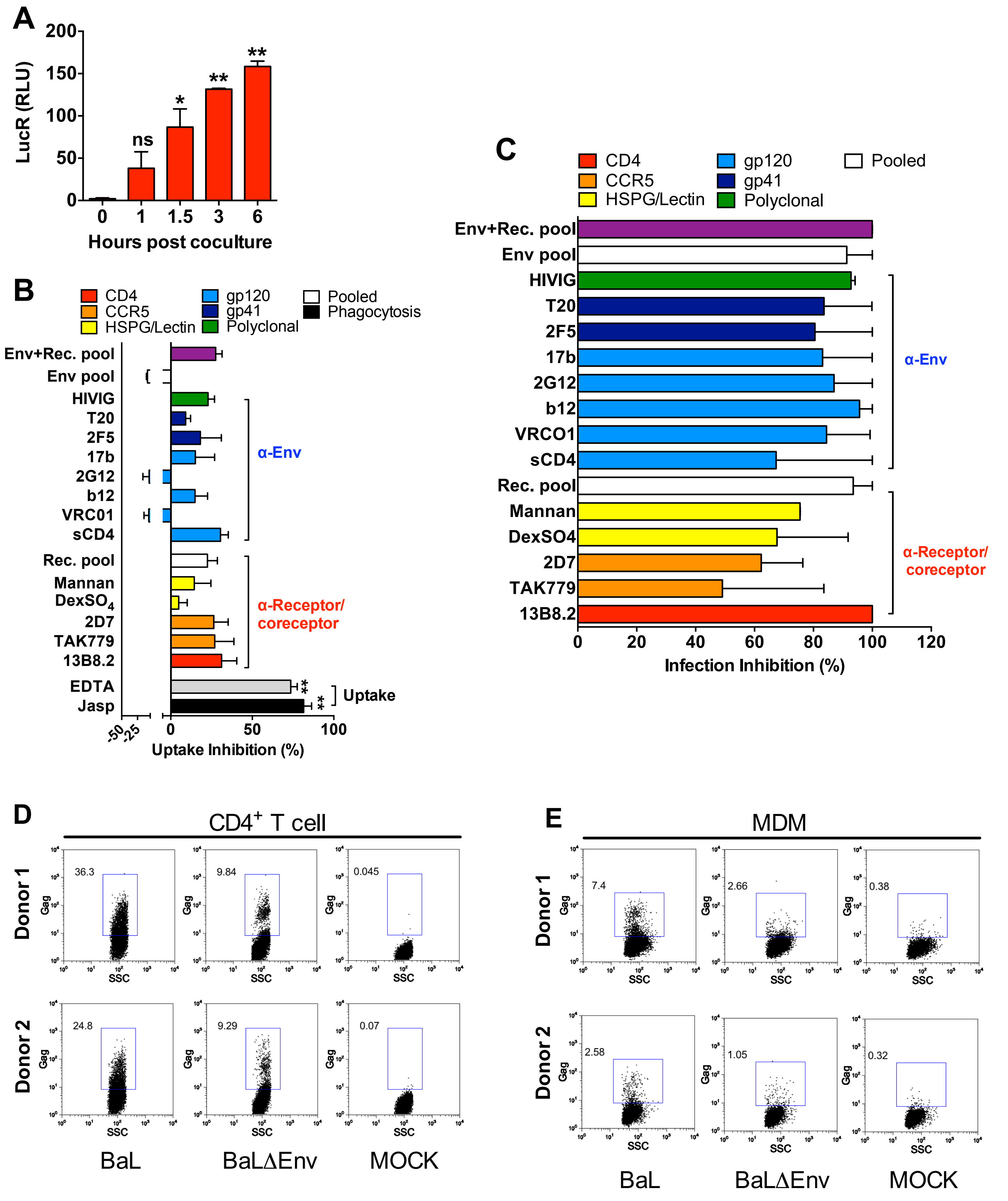
A
LucR (RLU)
Hours post coculture
ns
*
**
**
0
1
1.5
3
6
B
CD4
CCR5
HSPG/Lectin
gp120
gp41
Polyclonal
Pooled
Phagocytosis
Env+Rec. pool
Env pool
HIVIG
T20
2F5
17b
2G12
b12
VRC01
sCD4
Rec. pool
Mannan
DexSO4
2D7
TAK779
13B8.2
EDTA
Jasp
α-Env
α-Receptor/
coreceptor
Uptake
Uptake Inhibition (%)
C
CD4
CCR5
HSPG/Lectin
gp120
gp41
Polyclonal
Pooled
Env+Rec. pool
Env pool
HIVIG
T20
2F5
17b
2G12
b12
VRCO1
sCD4
Rec. pool
Mannan
DexSO4
2D7
TAK779
13B8.2
α-Env
α-Receptor/
coreceptor
Infection Inhibition (%)
D
CD4+ T cell
Donor 1
Donor 2
36.3
9.84
0.045
24.8
9.29
0.07
Gag
SSC
BaL
BaLΔEnv
MOCK
E
MDM
Donor 1
Donor 2
7.4
2.66
0.38
2.58
1.05
0.32
Gag
SSC
BaL
BaLΔEnv
MOCK

Figure S3. MDM uptake of HIV-1$^+$ T cells is resistant to inhibitors of Env-receptor interactions and is independent of Env (associated with Figures 2A - 2D, 3F + 5E).

(A + B) Experiments described in Figure 2 A + B were also carried out using luciferase reporter virus readout (also associated with Figures 3F and 5E). (A) HIV-$1_{BaL\text{-}LucR}$ -infected T cells prepared as described in Figure 1B and methods were incubated with MDM for the times shown, MDM were extensively washed to remove non-MDM-associated T cells, and cultures lyzed and assayed for luciferase activity. Bars represent mean luciferase signal for two independent donors each analyzed in triplicate. *p<0.05, **p<0.01 by one-way ANOVA with Dunnet's multiple comparison post-test comparing all columns to T = 0 hr initiation of coculture. (B) Uptake inhibition (%) of HIV-$1_{BaL\text{-}LucR}$-infected T cells by MDM by specific inhibitors relative to each inhibitor control. Bars represent mean % uptake inhibition + SEM from 4 – 9 independent donors in 4 independent experiments. **p<0.01 by one-sample *t*-test comparing each to a hypothetical value of 0 with Sidak's multiple comparison post-test. Inhibitor concentrations are described in supplementary methods. (C) Functional activity of the inhibitors was confirmed against cell-free HIV-1 (associated with Figures 2B, 3F +5E). 500 $TCID_{50}$ of HIV-$1_{BaL\text{-}LucR}$ was preincubated with inhibitors or vehicle control for 1 hr and added to MDM in triplicate for 48 hr prior to lysis and assay for luciferase activity. Results are expressed as % inhibition of infection + SEM for 2 independent donors in two independent experiments. (D + E) Purified CD4$^+$ T cells from 2 independent donors were synchronously infected using magnetofection with VSVg-pseudotyped HIV-1_{BaL} WT or HIV-$1_{BaL\Delta env}$ or mock infected, and used 48 hr post infection (associated with Figures 2C + D). (D) T cells were fixed, permeabilized and labeled for intracellular Gag (KC57-FITC) and 10^4 events analyzed by flow cytometry. Plots show % Gag$^+$ T cells for two donors (Donor 1 and Donor 2). (E) HIV-1$^+$ T cells prepared as above were cocultured with autologous MDM for 3 hr, MDM were extensively washed and lifted before analysis as in (D).

A

Acronym	Full name of Inhibitor	Mode of action	Concentration used	Reference
Jasp	Jasplakinolide	Polymerises and stabilizes F-actin by stimulating actin filament nucleation	5uM	[1]
ATP	ATP	Inhibits purinergic receptor $P2X_7$ engaging apoptotic cells	1mM	[2]
C2A	C2A	Competes for apoptotic cell phosphatidyl serine	100ug/ml	[3]
Calrec	Calreticulin	Misdirected to the surface of apoptotic cells as an 'eat me' signal	5ug/ml	[4,5]
CD36	CD36 mAb SM0	Blocks CD36 binding phosphatidyl serine on apoptotic cells	100ug/ml	[6]
CD44	Inhibitory CD44 mAB 515	Inhibits CD44 uptake of apoptotic material	50ug/ml	[7]
cRGD	Cyclized peptide cRGD	Competes integrin αvβ3-ligand binding	50uM	[3]
MFG	Anti-MFG-E8	Inhibits phosphatidyl serine-vitronectin receptor binding interactions	5ug/ml	[3]
PLS	Phospho-L-Serine	Competes for apoptotic cell surface phosphatidyl serine	1mM	[8]
rAV	rAnnexin V	Competes for phosphatidyl serine	6ug/2x10^5 cells	BD data sheet
RGDS	Peptide RGDS	Competes integrin αvβ3-ligand binding	50uM	[3,6]
TIM4	TIM4 polyclonal antibody	Inhibits TIM4 binding phosphatidyl serine on apoptotic cells	5ug/ml	[9,10]

B

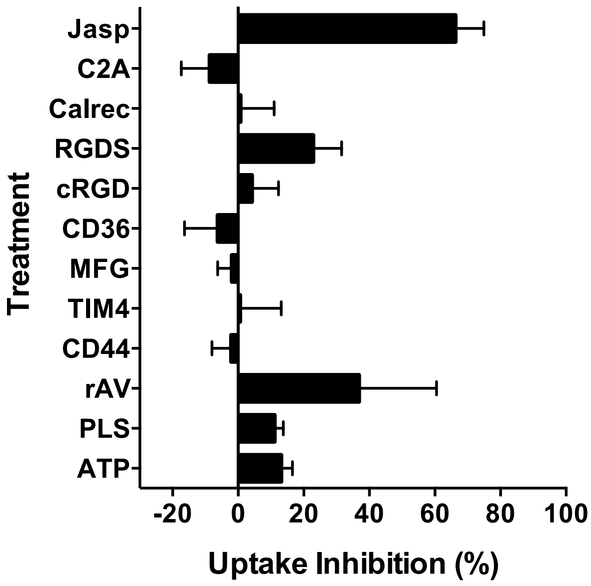

Figure S4. Published inhibitors of macrophage uptake of apoptotic/dying cells do not inhibit MDM uptake of HIV-1$^+$ T cells (associated with Figure 2B).

(A) Table of inhibitors published to interfere with phagocyte uptake of apoptotic cells tested for inhibition of HIV-1$^+$ T cell uptake by MDM. The inhibitor, mode of action, concentration used based upon the published active dose and relevant references are listed. Use of the inhibitors is described in supplementary methods. (B) Inhibition of HIV-1_{BaL}^+ T cell uptake by MDM using inhibitors of cell death recognition by phagocytes. Data represent the means + SEM of data from 2-6 independent donors. No significant effects on infected cell uptake were detected between any inhibitor using one-sample t-test comparing means to a hypothetical value of 0 with Sidak's multiple comparison post-test. Inhibition of uptake following jasplakinolide treatment is shown as a postive control.

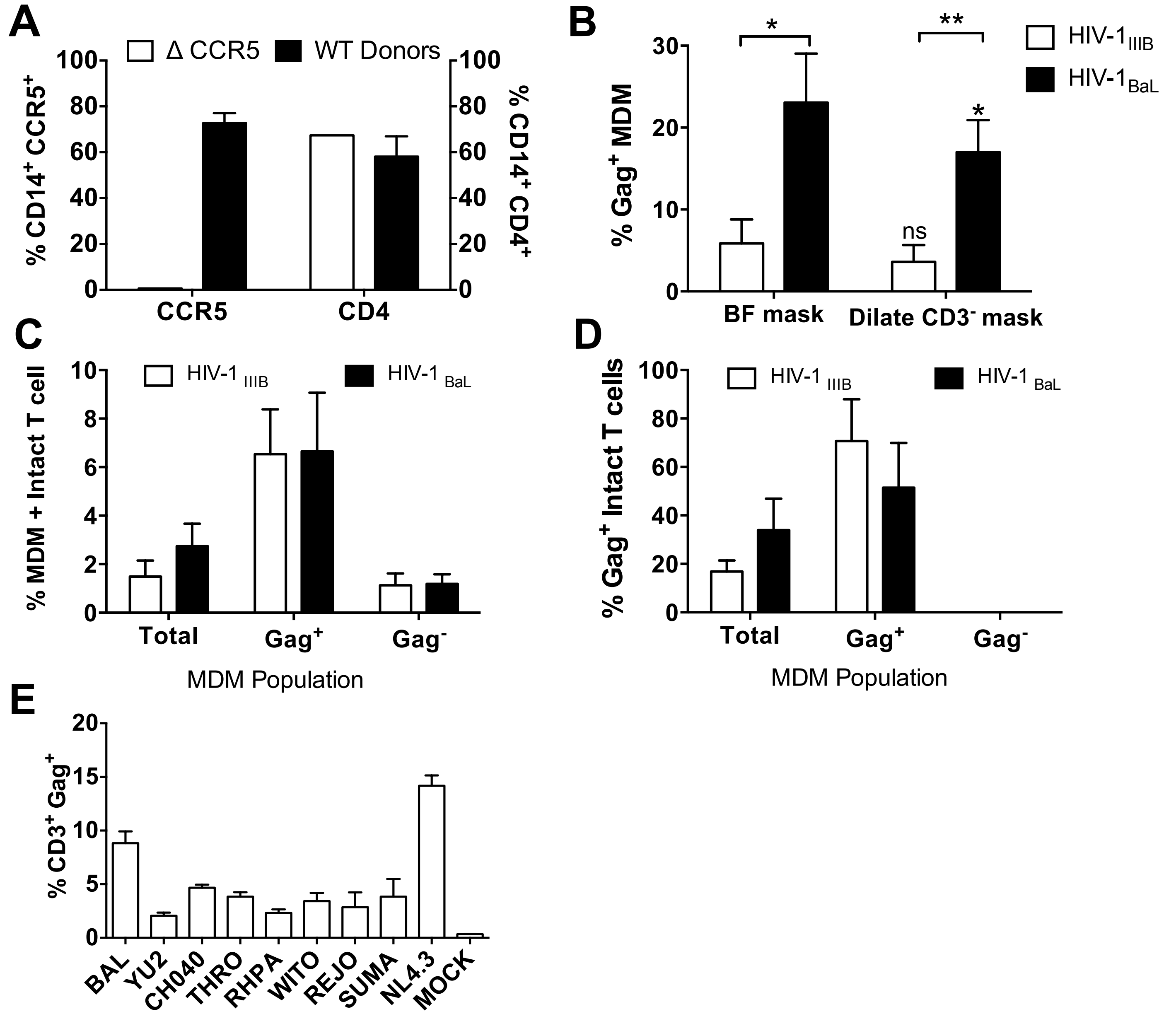

Figure S5. Phenotypic and infection controls and additional ImageStream analysis (associated with Figure 3G, 4A, 4C, 5A, 5B + 5C).
(A) CD4 and CCR5 expression in MDM derived from WT donors and a Δ32 CCR5 heterozygote donor (associated with Figure 3G). MDM derived as described in methods from five WT and one Δ32 CCR5 homozygous donor were lifted and labelled for CD14, CCR5 and CD4 then analyzed by flow cytometry. MDM were selected based on size/granularity and CD14 labelling. The percentage of $CD14^+$ MDM that were $CCR5^+$ (left axis) or $CD4^+$ (right axis) was determined. White bars represent % marker positive MDM from a single Δ32 CCR5 homozygous donor, black bars represent mean % marker positive MDM from five independent donors + SEM. (B, C + D). ImageStream analysis of cytoplasmic Gag and intact T cells within MDM (associated with Figures 4A + 4C). As described in Figure 4, MDM were cocultured with $CD4^+$ T cells infected with HIV-1_{IIB} or HIV-1_{BaL} for 3 hr, washed extensively and maintained in culture for 6 days. MDM were lifted, fixed and stained for HIV-1 Gag and CD3. (B) Cytoplasmic Gag staining of MDM. MDM were analysed for Gag staining within either a total MDM mask based on brightfield (BF mask) or the BF MDM mask excluding the $CD3^+$ regions ($CD3^-$ Gag^+) based on a restricted CD3 mask (Dilate $CD3^-$ Mask). The latter mask represents cytoplasmic Gag staining. Bars represent mean + SEM. *p<0.05, **p<0.01 by paired Student's t-test for both mask sets comparing HIV-1_{BaL} (white) to HIV-1_{IIB} (black) "ns" p>0.05, *p<0.05 by one sample t-test comparing % Gag^+ within the 'Dilate $CD3^-$ mask' to a hypothetical value of 0 with Sidak's multiple comparison post-test. (C) MDM were gated on those containing a bright CD3 stain with an area of >100 pixels (corresponding to 25 em^2) and a round shape (aspect ratio >0.7) for MDM infected with HIV-1_{IIIB} (white) or HIV-1_{BaL} (black). Data shown are the mean % of MDM containing intact T cells based on the previous parameters + SEM for the total MDM population, Gag^+ MDM and Gag^- MDM. *Continued on next page.*

Figure S5 Cont. (D) Intact T cells within MDM in (C) were analysed for Gag expresion. The % of such T cells that were Gag$^+$ are shown. n=4 independent donors from 2 independent experiments. E) T cell infection with luciferase reporter IMC expressing T/F virus Envs (associated with Figures 5A, 5B + 5C). CD4$^+$ T cells prepared as described in methods were infected with HIV-1$_{NL4.3\Delta env\text{-}LucR}$ carrying Envs from either two macrophage-tropic viruses (BaL, YU2), six T/F viruses (CH040, THR0, RHPA, WITO, REJO, SUMA), X4 non-macrophage-tropic virus (NL4.3) or mock infected. Cells were fixed, permeabilized and labeled for CD3 and Gag. Bars represent mean Gag$^+$ T cells + SEM from 8 – 13 donors analyzed per *env* clone.

SUPPLEMENTAL LEGENDS

MOVIES S1 - S3: Live cell MDM uptake of HIV-1^{+} T cells

Movies S1 and S2: Jurkat-CCR5 T cells infected for 7-10 days with (Movie S1) X4 HIV-1$_{NL4.3\text{-}eGFP}$ or (Movie S2) R5 HIV-1$_{CH077\text{-}mCherry}$ were mixed with adherent MDM and immediately imaged. (Movie S3) Primary activated CD4^{+} T cells synchronously infected with HIV-1 CH077$_{mCherry}$ were mixed with autologous MDM and immediately imaged. Time-lapse imaging was over approximately 2 hr in a conditioned atmosphere with an Axiovert 200 microscope with Axiovision MRm CCD camera and Colibri illumination.

SUPPLEMENTAL EXPERIMENTAL PROTOCOLS

Live cell video microscopy (Movies S1, S2 and S3 and Figure 1A)

Jurkat.Tat.R5 or primary $CD4^+$ T cells infected with HIV-$1_{CH077mCherry}$ or HIV-$1_{NL4\text{-}3\ GFP}$ were washed to remove cell-free virus, added to MDM (1:1) cultured on ibidi μ-Slide I (Thistle Scientific), and immediately imaged by time-lapse with a 40 x objective using an Axiovert 200 microscope with Axiovision MRm CCD camera and Colibri illumination (Zeiss Inc).

Cell-free infection route comparison (Figure S2A, B + C)

$CD4^+$ T cells infected with HIV-$1_{BaL\text{-}luc}$ were washed to remove cell-free virus and resuspended at 3 concentrations (corresponding to MDM:T cell ratios of 1:2, 1:1 and 2:1). $CD4^+$ T cells were cultured for 6 hr, supernatants collected and added to MDM as cell-free virus (CF). Corresponding T cells were washed to remove any residual cell-free virus, resuspended at the same concentration and added to MDM either cell-to-cell (CTC) or through a transwell (TW). Cell preparations / supernatants were cultured with MDM for a further 6 hr. MDM were washed gently to remove virus, or extensively to remove T cells and cultured for a further 3 days. MDM were lysed and analyzed for luciferase expression as described.

MDM functional analyses (Figure S2D, E + F)

Cytotoxicity of uptake-inhibiting drugs (jasplakinolide, EIPA) was assessed on MDM with the MTS metabolic activity assay (Promega). Functional activity was confirmed by a flow-cytometry based phagocytosis or macropinocytosis assay by quantifying fluorescent bead uptake (Fluoresbrite YG carboxylated microspheres 6.0 μm, Polysciences) or 150 kDa FITC-dextran (Sigma) uptake as a percentage of vehicle control-treated MDM after lifting.

Inhibition of HIV-1$^+$ T cell uptake studies (Figures S3A-C and S4)

Inhibitors of MDM uptake were: jasplakinolide (5 μM, Molecular Probes); 5-N-ethyl-N-isopropyl amiloride (EIPA, 50 μM, Sigma-Aldrich), EDTA (2 mM, Sigma-Aldrich). Inhibitors of HIV-1 Env-receptor interactions were as follows: dextran sulfate (10 μg/mL, Sigma-Aldrich); mannan (20 μg/mL, Sigma-Aldrich); VRC01 (10 μg/mL, kindly provided by J. Mascola); IgG1b12 Fab (10 μg/mL, kindly provided by D. Burton, Scripps Research Institute, USA); 2G12 (10 μg/mL); 17b (10 μg/mL); 2F5 (10 μg/mL); HIVIG (10 μg/mL, NIH AIDS Reagent Repository, USA); T20 (7.5 μM); 13B.8.2, CD4-specific gp120 blocking mAb (10 μg/mL Beckman-Coulter); sCD4 (20 μg/mL, IAVI); Tak-779 (500 nM, CFAR); 2D7 CCR5-specific gp120-blocking mAb (10μg/mL, CFAR); anti-human IgG1 isotype control (10 μg/mL, kindly provided by H. Waldman). 17b neutralization was carried out in the presence of 1 μg/mL sCD4 to induce the CD4i site. Relevant isotype controls were used at matched concentrations. Antagonists of dead and dying cell uptake listed in Figure S4 were used at published functional concentrations, and are referenced below. For experiments using anti-HIV-1 inhibitors (neutralizing antibodies and CD4 binding site and gp41 ligands) or inhibitors of apoptotic cell uptake that ligate molecules on the T cell, HIV-1$^+$ T cells were incubated for 1 hr with double the final concentration of inhibitor before addition to MDM in an equal volume of medium. For inhibitors of viral receptors, inhibitors of receptors of apoptotic cell uptake and EDTA, MDM were incubated with double the concentration of inhibitor for 1h before addition of HIV-1$^+$ T cells in an equal volume of medium. For inhibition of cytoskeletal remodeling with jasplakinolide, MDM were incubated with the final concentration for 1 hr followed by washing prior to coculture with HIV-1$^+$ T cells. MDM-HIV-1$^+$ T cell cocultures were then maintained for 30-90 mins prior to washing 3-4 times in PBS/ 5nm EDTA to remove

unattached/engulfed T cells, followed by MDM processing for vDNA PCR or luciferase assay. For Figure S4C, 500 $TCID_{50}$ of primary $CD4^+$ T cell-derived virus supernatant were incubated with the inhibitors shown for 1 hr and added to MDM in triplicate for 48 hr prior to lysis and assay for luciferase activity as described.

ImageStream gating strategy (Figure S5B, C + D)

$CD4^+$ T cells were infected with either HIV-1_{BaL} or HIV-1_{IIIB} and cocultured with MDM and washed to remove T cells as described for Figure 4A and 4C. Briefly, on Day 6 post-coculture MDM were washed, fixed, stained for CD3 and Gag and analyzed by ImageStream^{TM}. To determine the % of MDM containing cytoplasmic Gag^+, a mask was created in IDEAS to define the whole MDM area based on brightfield as a region of interest (BF mask). Separately, a CD3 mask was generated based on CD3 stain and dilated by 4 pixels. The CD3 mask was subtracted from the BF mask to define the MDM cytoplasm (Dilate $CD3^-$ mask) and the % of MDM that were Gag^+ within this mask were determined. To determine the % of MDM containing intact $CD3^+$ T cells, a stepwise gating strategy was established. Firstly, MDM containing a CD3 stain were gated based on CD3 intensity. Then the size of the CD3 stain was determined and MDMs containing CD3 stain of over 100 pixels in area (>$25cm^2$) were selected. Finally, the CD3 area within the MDM was analyzed for aspect ratio (roundness) indicative of an intact T cell rather than debris (aspect ratio >0.7). The final gate defined MDMs that contain bright CD3 stains, which are round and have a size indicative of a $CD4^+$ T cell. To determine the % of these $CD3^+$ areas that were Gag^+, a mask was defined based on CD3 intensity and Gag intensity determined within that area.

SUPPLEMENTAL REFERENCES

Associated with Figure S4A

1. Bubb, M., et al., *Jasplakinolide, a cytotoxic natural product, induces actin polymerisation and competitively inhibits the binding of phalloidin to F-actin.* J. Biol. Chem., 1994. **269**: p. 14869-14871.
2. Gu, B.J., et al., *P2X(7) is a scavenger receptor for apoptotic cells in the absence of its ligand, extracellular ATP.* Journal of immunology, 2011. **187**(5): p. 2365-75.
3. Neher, J.J., et al., *Inhibition of microglial phagocytosis is sufficient to prevent inflammatory neuronal death.* Journal of immunology, 2011. **186**(8): p. 4973-83.
4. Obeid, M., et al., *Calreticulin exposure dictates the immunogenicity of cancer cell death.* Nature medicine, 2007. **13**(1): p. 54-61.
5. Gardai, S.J., et al., *Cell-surface calreticulin initiates clearance of viable or apoptotic cells through trans-activation of LRP on the phagocyte.* Cell, 2005. **123**(2): p. 321-34.
6. Fadok, V.A., et al., *CD36 is required for phagocytosis of apoptotic cells by human macrophages that use either a phosphatidylserine receptor or the vitronectin receptor (alpha v beta 3).* Journal of immunology, 1998. **161**(11): p. 6250-7.
7. Frleta, D., et al., *HIV-1 infection-induced apoptotic microparticles inhibit human DCs via CD44.* The Journal of clinical investigation, 2012. **122**(12): p. 4685-97.
8. Kurosaka, K., et al., *Silent cleanup of very early apoptotic cells by macrophages.* Journal of immunology, 2003. **171**(9): p. 4672-9.
9. Santiago, C., et al., *Structures of T cell immunoglobulin mucin protein 4 show a metal-Ion-dependent ligand binding site where phosphatidylserine binds.* Immunity, 2007. **27**(6): p. 941-51.
10. Kobayashi, N., et al., *TIM-1 and TIM-4 glycoproteins bind phosphatidylserine and mediate uptake of apoptotic cells.* Immunity, 2007. **27**(6): p. 927-40.
